# Effects of Vitamin D Supplementation on Bone Turnover and Bone Mineral Density in Healthy Men: A Post-Hoc Analysis of a Randomized Controlled Trial

**DOI:** 10.3390/nu11040731

**Published:** 2019-03-29

**Authors:** Elisabeth Lerchbaum, Christian Trummer, Verena Theiler-Schwetz, Martina Kollmann, Monika Wölfler, Stefan Pilz, Barbara Obermayer-Pietsch

**Affiliations:** 1 Department of Internal Medicine, Division of Endocrinology and Diabetology, Medical University of Graz, Auenbruggerplatz 15, 8036 Graz, Austria; christian.trummer@medunigraz.at (C.T.); verena.schwetz@medunigraz.at (V.T.-S.); stefan.pilz@medunigraz.at (S.P.); barbara.obermayer@medunigraz.at (B.O.-P.); 2 Department of Obstetrics and Gynecology, Division of Gynecological Endocrinology and Reproductive Medicine, Medical University of Graz, Auenbruggerplatz 14, 8036 Graz, Austria; martina.kollmann@medunigraz.at (M.K.); monika.woelfler@medunigraz.at (M.W.)

**Keywords:** vitamin D, randomized-controlled trial, bone turnover markers, bone mineral density

## Abstract

Vitamin D is well known for its effects on calcium and mineral metabolism. However, vitamin D effects on bone turnover markers (BTMs), which are used together with bone mineral density (BMD) to evaluate bone health, are less clear. We therefore examined vitamin D effects on BTMs (beta-cross laps (CTX) and osteocalcin (OC)) and BMD in a post-hoc analysis of a randomized controlled trial (RCT). This is a post-hoc analysis of the Graz Vitamin D&TT-RCT, a single-center, double-blind, randomized placebo-controlled trial conducted between December 2012 and November 2017 at the endocrine outpatient clinic at the Medical University of Graz, Austria. A total of 200 healthy men with serum 25-hydroxyvitamin D (25(OH)D) levels <75 nmol/L participated in the trial. Subjects were randomized to receive 20,000 IU of vitamin D3/week (*n* = 100) or placebo (*n* = 100) for 12 weeks. Outcome measures were BTMs, BMD, and trabecular bone score (TBS). A total of 192 men (mean age and 25(OH)D: 43 (±13) years and 54.9 (±18.3) nmol/L, respectively) completed the study. We found no significant treatment effect on BTMs, BMD, or TBS (*p* > 0.05 for all). In middle-aged healthy men, vitamin D treatment for 12 weeks had no significant effect on BTMs or BMD.

## 1. Introduction

Vitamin D is a steroid hormone that is well known for its effects on calcium and mineral metabolism [1]. Vitamin D deficiency leads to impaired mineralization of bone due to an inefficient absorption of dietary calcium and phosphorus and is associated with an increase in parathyroid hormone (PTH) levels [2,3]. Clinical symptoms of severe vitamin D deficiency as reflected by 25-hydroxyvitamin D (25(OH)D) levels <25 nmol/L manifest as osteomalacia in adults (existing bones) and rickets in children (growing bones) [2]. Less severe vitamin D deficiency causes secondary hyperparathyroidism and increases bone turnover and bone loss [4]. Therefore, vitamin D has long been recommended to prevent or treat osteoporosis [5]. This was also due to the fact that early evidence suggested benefits for musculoskeletal health including an increase in bone mineral density (BMD) as well as a possible prevention of falls and fractures [3]. However, more recent reviews and meta-analyses revealed neutral or only small favorable effects on falls and fractures [5,6,7]. Further, a recent meta-analysis including 41 randomized controlled trials (RCTs) on BMD found no clinically meaningful effect of vitamin D supplementation on BMD [6].

Various systemic and local factors, as well as nutritional factors such as calcium and vitamin D, regulate bone remodeling. In addition to BMD, bone turnover markers (BTMs) such as beta-crosslaps (CTX), a marker of bone resorption, or osteocalcin (OC), a marker of bone formation, are used to evaluate bone health [8]. Whereas structural bone changes reflected by a low or decreasing BMD may take some time to develop, changes in BTMs develop faster and are therefore used to clinically evaluate the response to treatment in osteoporosis [9]. However, existing evidence on vitamin D effects on BTMs is sparse and revealed conflicting results [10,11,12]. Therefore, it has been suggested that more research is needed regarding the relationship between changes in vitamin D and BTM levels [2].

Given these inconsistent results on vitamin D effects on BTMs, we performed a post-hoc analysis of the Graz Vitamin D&TT-RCT, a RCT including 100 men with normal total testosterone (TT) concentrations and 100 men with low TT concentrations. The trial was conducted in healthy men with 25(OH)D concentrations <75 nmol/L in order to evaluate vitamin D effects on testosterone concentrations as the primary outcome [13,14]. In this post-hoc analysis, we investigated the effect of vitamin D versus placebo on BTMs (CTX and OC), BMD, as well as on trabecular bone score (TBS).

## 2. Materials and Methods

This study is a post-hoc analysis of the Graz Vitamin D&TT-RCT, a single-center, double-blind, placebo-controlled, parallel-group study performed at the Medical University of Graz, Austria. The trial was designed to investigate the effect of 12 weeks of vitamin D supplementation on TT levels in men. The methods and study design have been published in detail previously [13,14]. The design, conduction, and publication of this study adhere to the recommendations of the CONSORT Statement (http://www.consort-statement.org/). The trial was registered at http://www.clinicaltrialsregister.eu (EudraCT number, 2011-003575-11) and at clinicaltrials.gov (ClinicalTrials.gov Identifier NCT01748370). The study protocol was approved by the ethics committee of the Medical University of Graz (EK 23-513 ex 10/11) and written informed consent was obtained from each participant before entering the study.

### 2.1. Subjects

Eligible study participants were men aged ≥18 and <70 years with 25-hydroxyvitamin D (25(OH)D) levels <75 nmol/L. As published previously [13,14], exclusion criteria were hypercalcemia (defined as a serum calcium >2.65 mmol/L), oral or transdermal testosterone supplementation in the last 2 months before entering the study, intramuscular testosterone supplementation 6 months before entering the study, regular intake of vitamin D supplements before study entry, osteoporosis, chronic diseases (such as diabetes mellitus), thyroid disease, endocrine disturbances in need of treatment (such as pituitary disorders), history of hypogonadism or known diseases associated with hypogonadism (except obesity) or diseases known to interfere with vitamin D intake or sensitive to vitamin D intake (including inflammatory diseases with granuloma such as sarcoidosis, tuberculosis, Wegener’s granulomatosis; including other forms of vasculitis and inflammatory bowel diseases), intake of medication influencing metabolic or endocrine parameters (insulin sensitizers, insulin, or glucocorticoids) in the last 3 months before study entry; PSA >4 ng/mL (or >3 ng/mL in men at high risk for prostate cancer), palpable prostate nodule or induration, hematocrit >50%, untreated severe obstructive sleep apnea, severe lower urinary tract symptoms, uncontrolled or poorly controlled heart failure, a history of prostate cancer, breast cancer, orchidectomy, and chromosomal disorders (e.g., Klinefelter Syndrome). Men were recruited from the outpatient clinic of the Department of Internal Medicine, Division of Endocrinology and Diabetology, and the outpatient clinic of the Department of Urology, Medical University of Graz, Austria, as well as from male hospital staff and male family members of hospital staff. Men were informed about the trial either by a conversation in the outpatient clinic, by written information posted in the respective outpatient clinics, or by a telephone call. All patients were informed that participation in the study is voluntary and that refusal to participate as well as stopping at any time without giving reasons, without any consequences is possible. Written informed consent was obtained before carrying out any study related procedures from all subjects who participated in the study.

### 2.2. Intervention

Subjects were allocated to the vitamin D or placebo group according to a computer-generated randomization list using a ratio of 1:1. Study medication was placed into numbered bottles according to this computer-generated randomization list. Randomization procedures were conducted using a web-based software (http://www.randomizer.at/) with GCP compliance as confirmed by the Austrian Agency for Health and Food Safety (AGES).

The treatment group received an oral dose of 20,000 IU vitamin D weekly (equivalent to 2857 IU/day) as 50 oily drops weekly (Oleovit D3-drops; Fresenius Kabi Austria GmbH, Linz) for 12 weeks and the placebo group received 50 oily drops without vitamin D for 12 weeks. Placebo oil contained the same oil as Oleovit D3-drops (without vitamin D content) and was delivered by Fresenius Kabi Austria GmbH, Linz. All investigators who enrolled participants, collected data, and assigned intervention were masked to participant allocation.

To improve and verify compliance, patients were asked to return the study medication bottles (full as well as empty bottles) at study end (visit 3).

### 2.3. Outcome Measures

This is a post-hoc analysis of the Graz Vitamin D&TT-RCT investigating vitamin D effects compared to placebo on BTMs (CTX and OC), BMD (total body, lumbar spine, total hip, and femoral neck), and trabecular bone score (TBS).

### 2.4. Procedures

Basal blood samples for 25(OH)D, PTH, TT, CTX, OC, and the remaining parameters were collected between 8.00 and 9.00 a.m. after an overnight fast. 25(OH)D and TT measured by immunoassays were used for evaluation of inclusion criteria. Biobanking of remaining blood samples was performed by freezing and storing at −80 °C until analysis. Serum levels of 25(OH)D and TT were additionally measured by well-adjusted Isotope-Dilution Liquid Chromatography Tandem Mass Spectrometry (ID-LC-MS/MS) methods in 2018 [15,16]. Only 25(OH)D levels measured by ID-LC-MS/MS are used for all statistical analyses in the manuscript. OC (intra- and interassay CV, 0.5% and 1.4%, respectively) was measured by electrochemiluminescence immunoassay (Roche Diagnostics, Mannheim, Germany) according to the manufacturer’s instructions. CTX (intra- and inter-assay CV, 2.0% and 4.2%, respectively) levels were measured by electrochemiluminescence immunoassay (Cobas, Roche Diagnostics, Mannheim, Germany). BMD measurements were performed at the lumbar spine and femoral neck (Lunar iDXA^®^, GE). Least significant changes of BMD measured at the lumbar spine, total hip, and femoral neck are 2.29%, 3.75%, and 3.82%, respectively. Two investigators performed all analyses. The equipment was calibrated each day using a standardized phantom to detect drifts in measurements, and equipment servicing was performed regularly.

### 2.5. Statistical Analyses

Details on sample size calculation have been published previously [13]. Continuous data are presented as median with interquartile range. The distribution of data was analyzed by descriptive statistics and Kolmogorov–Smirnov test. Skewed variables were log transformed and rechecked for normal distribution. Student’s *t*-test was used for comparisons of baseline characteristics between groups. Analyses of outcome variables were performed according to the intention-to-treat principle and inclusion of all participants with baseline and follow-up values. Analysis of covariance with adjustments for baseline values was applied to test for differences in the outcome variables between the treatment and the placebo group at study end. We performed subgroup analyses in men with 25(OH)D levels <50 nmol/L, in men with 25(OH)D levels lower and higher than 40 nmol/L, in men with a body mass index (BMI) lower and higher than the median BMI (26.4 kg/m^2^), in men with a BMI lower and higher than 25 kg/m^2^, and in men with baseline TT levels lower and higher than 10.41 nmol/L. To study seasonal variation, we subdivided the year into 3-month measurement periods: January–March (season 1); April–June (season 2); July–September (season 3); and October–December (season 4) to address the seasonal changes in availability of sunlight. Seasonal variation of 25(OH)D, PTH, and BTMs was analyzed by ANOVA. Paired student’s *t*-test was used for comparisons of 25(OH)D levels at baseline and follow-up in the placebo group. All statistical procedures were performed with SPSS version 23 (SPSS Inc., Chicago, IL, USA). A *p*-value <0.05 was considered statistically significant.

## 3. Results

We took blood samples from approximately 1100 men and analyzed 25(OH)D concentrations and TT concentrations (participant flow charts have been published previously [13,14]). Two-hundred men who met all inclusion as well as no exclusion criteria and gave their written informed consent were randomized and enrolled in the study. The first subject was randomized in December 2012 and the last follow-up was performed in November 2017. Baseline characteristics of all study participants are shown in Table 1. Median *t*-scores measured at total body, lumbar spine, total hip, and femoral neck were 1.0 (0.3–1.6), 0.0 (−1.0 to 0.7), −0.4 (−1.0 to 0.4), and −0.1 (−0.7 to 0.6), respectively. No participant took any osteoporosis drug such as bisphosphonates before or during the study. We observed no significant difference in baseline characteristics between the vitamin D and the placebo group. The mean overall treatment period was 86 ± 7 days in the vitamin D and 86 ± 7 days in the placebo group (*p* = 0.422). A total of 192 men completed the study and were analyzed for outcome measures.

### 3.1. Outcome Parameters

We show results of analyses of outcomes measures in Table 2. We found no significant treatment effect on BTMs, BMD, or TBS.

### 3.2. Subgroup Analyses

#### 3.2.1. Vitamin D

In unadjusted analyses, we observed significantly lower BMD at the femoral neck (*p* = 0.003) in men with baseline 25(OH)D levels ≥50 nmol/L (*n* = 115) compared to men with lower baseline 25(OH)D levels (*n* = 85), whereas BTMs, total body BMD, lumbar spine BMD, total hip, TBS, as well as PTH levels were similar in both groups (*p* > 0.05 for all).

We observed no significant vitamin D effects on BTMs, BMD, or TBS in men with 25(OH)D levels <50 nmol/L (*p* > 0.05 for all). As only 15 men presented with 25(OH)D levels <30 nmol/L, we could not perform an adequate analysis in this subgroup.

In men with baseline 25(OH)D levels <40 nmol/L (*n* = 35), we found a significant vitamin D effect on 25(OH)D levels (Table 3) whereas we observed no significant vitamin D effect on PTH (Table 3) or outcome measures (*p* > 0.05 for all). In men with baseline 25(OH)D levels ≥40 nmol/L, we found a significant vitamin D effect on 25(OH)D and PTH levels (Table 3) whereas we observed no significant vitamin D effect on our outcome measures (*p* > 0.05 for all).

#### 3.2.2. BMI

Subjects with a BMI <26.4 kg/m^2^ (*n* = 100) had significantly higher CTX and OC, whereas total body BMD as well as the BMD at the lumbar spine and total hip were significantly lower compared to men with a higher BMI (*n* = 100) (*p* < 0.05 for all.

In subjects with a BMI <26.4 kg/m^2^, vitamin D had a significant treatment effect on 25(OH)D, PTH, and total body BMD (Table 4).

In subjects with a BMI ≥26.4 kg/m^2^, we observed a significant treatment effect on 25(OH)D levels whereas vitamin D treatment had no significant effect on PTH and all outcome parameters (data not shown).

When men were stratified by a BMI of 25 kg/m^2^, we observed again a significant treatment effect on total body BMD (0.012 (0.002 to 0.021) g/cm^2^, *p* = 0.017), 25(OH)D (Table 3), and PTH (Table 3) in normal weight men (*n* = 65) but no significant effect (except on 25(OH)D levels) in overweight/obese men (*n* = 135, Table 3).

#### 3.2.3. Testosterone

In subjects with baseline TT levels <10.41 nmol/L, we observed a significant vitamin D effect on 25(OH)D levels (Table 3), whereas we found no significant vitamin D effect on PTH levels (Table 3) or outcome measures (*p* > 0.05 for all, data not shown). In subjects with baseline TT levels ≥10.41 nmol/L, we observed a significant vitamin D effect on 25(OH)D and PTH levels (Table 3) but no significant effect on any outcome parameter (*p* < 0.05 for all).

#### 3.2.4. Seasonality

The majority of subjects were recruited in season 1 and 2 (Table 1). When stratified by season, we observed a significant difference in 25(OH)D (highest levels in season 3, lowest levels in season 1; *p* < 0.001) and PTH levels (highest levels in season 1, lowest levels in season 2; *p* = 0.006) as well as a trend towards different OC levels (highest levels in season 4, lowest levels in season 3; *p* = 0.058). No significant seasonal variation was seen for BCTX levels (*p* > 0.05).

Individual changes in 25(OH)D levels in the placebo group are shown in supplemental Figure S1. In the placebo group, we found a significant increase in 25(OH)D levels in all study participants as well as in all subgroup analyses stratified by 25(OH)D levels (>40 nmol/L), BMI (below and above 25 kg/m^2^), and TT levels (lower and higher than 10.41 nmol/L) except for subjects with baseline 25(OH)D levels <40 nmol/L (*p* = 0.051).

## 4. Discussion

In this RCT, in healthy middle-aged men with 25(OH)D levels <75 nmol/L at baseline, vitamin D treatment had no significant effect on BTMs or BMD. We observed, however, an increase in total body BMD in normal weight men.

Our results demonstrating no effect on BTMs are in line with previous studies performed in hypertensive patients [12], healthy postmenopausal women [17], healthy young and elderly adults [18], as well as healthy obese men and women [19]. In contrast, a decrease in bone formation markers but no effect on bone resorption has been observed in healthy white men receiving vitamin D during winter time [11]. Results from the longitudinal aging study Amsterdam suggested that a significant vitamin D effect on BTMs might be limited to subjects with low 25(OH)D levels [20]. We failed, however, to find significant vitamin D effects on CTX and OC in men with low 25(OHD levels (<50 nmol/L, *n* = 85)), respectively.

Further, there was no significant effect of vitamin D treatment on BMD, which is in line with a recent meta-analysis [6]. In contrast, Larsen et al. [15] found a small, but significant positive effect of five years of vitamin D supplementation at a dose of 20,000 IU weekly on femoral neck BMD in males with prediabetes. In this context, it should also be noted that subgroup analyses of RCTs suggested that improvements of BMD by vitamin D supplementation may be observed only in individuals with 25(OH)D levels ≤30 nmol/L with no significant effect at higher 25(OH)D levels [16,21]. However, we found no significant vitamin D effect in a subgroup analysis of men with 25(OH)D levels <50 nmol/L. As only 15 men presented with 25(OH)D levels <30 nmol/L, we did not perform further analyses in this subgroup and we therefore cannot exclude significant vitamin D effects in this subgroup. It should also be noted that duration of treatment (12 weeks) may be too short to exert a significant effect on BMD. Nevertheless, in a RCT in 81 postmenopausal women with 25(OH)D concentrations below 50 nmol/L, high-resolution peripheral quantitative computed tomography (HRpQCT) scans showed improved bone strength and trabecular thickness in the tibia, and increased BMD in the trochanter and femoral neck with no effect on DXA BMD measures after three months of vitamin D_3_ supplementation at a dose of 2800 IU daily [17].

There is an ongoing scientific debate regarding optimal vitamin D levels required for musculoskeletal health. We used a 25(OH)D level of <75 nmol/L as inclusion criteria as the Endocrine Society Clinical Practice Guideline [22], a major vitamin D guideline for patient care, suggested a 25(OH)D level of >75 nmol/L as optimal. This cut-off was based on studies showing an increased intestinal calcium absorption and a decreased level of circulating PTH when 25(OH)D values were >75 nmol/L [22,23]. Similarly, Bischoff-Ferrari [24] suggested that for the improvement of endpoints such as BMD, incident falls, and fractures, the most appropriate serum 25(OH)D level is >75 nmol/L. However, it should be pointed out that several other vitamin D guidelines suggested lower optimal vitamin D level [25,26,27,28,29]. The Institute of Medicine (IOM) report concluded that there is no additional benefit of achieving serum 25(OH)D concentrations of 75 nmol/L when compared to 50 nmol/L [25]. Further, the Italian Association of Clinical Endocrinologists (AME) and Italian Chapter of the American Association of Clinical Endocrinologists (AACE) Position Statement regarding the Clinical Management of Vitamin D Deficiency in Adults established that a 25(OH)D levels of 50 nmol/L can be considered appropriate in the general population, while 75 nmol/L should be considered in categories at risk such as osteopenia, osteoporosis, or obesity [27]. In light of these controversial scientific discussions, we cannot exclude that the lack of significant results in our trial is related to the relatively high baseline 25(OH)D levels.

BMI is a major determinant of BMD [30] and the effect of vitamin D supplementation is related to BMI, as obese subjects might require higher doses of vitamin D [22,31]. In detail, when obese and non-obese adults receive similar doses of vitamin D, they were able to raise their blood levels of vitamin D by no more than 50% compared with non-obese adults [22]. Consequently, the Endocrine Society Clinical Practice Guideline suggests 2–3 times higher vitamin D doses in obese subjects [22]. To address this issue, we performed subgroup analyses of normal weight and overweight/obese subjects. Interestingly, we observed a significant vitamin D effect on PTH levels in normal weight men whereas no significant effect was observed in men with higher BMI. Correspondingly, we found a significant positive vitamin D effect on total body BMD in normal weight men but not in men with higher BMI. We are, however, aware of the fact that multiple testing is an issue in this post-hoc analysis. Our results are no longer significant when our analyses are adjusted for multiple testing (i.e., Bonferroni correction) by dividing the *p*-value for statistical significance by the number of tests. Nevertheless, our results might contribute to the existing evidence demonstrating that higher vitamin D doses might be required in overweight/obese subjects to exert a beneficial effect on PTH levels and maybe also on bone health.

Our study has several limitations that should be noted. First, as we investigated men with relatively high 25(OH)D levels at baseline, we cannot exclude significant effects on BTMs or BMD in men with lower 25(OH)D levels. Indeed, our subgroup analyses involving men with low 25(OH)D levels at baseline are limited by the relatively low sample size. As vitamin D supplementation reduced PTH levels only in normal weight men, we cannot exclude significant vitamin D effects on our outcome measures including BTMs in a cohort of normal weight men. Further, as the study duration was 12 weeks for our study participants, we were not able to analyze any long-term effects on outcome measures. The study duration of 12 weeks might be too short to detect significant effects on BMD and we did not have HRpQCT data or other relevant bone markers such as bone-specific alkaline phosphatase. As we recruited the majority of subjects in season 1 and 2, we observed a significant increase in 25(OH)D levels not only in the vitamin D but also in the placebo group. The strengths of our study include the design as an RCT as well as the use of a state-of-the-art methods for measuring 25(OH)D levels. Further, vitamin D treatment was effective as reflected by the increase in 25(OH)D levels and the decrease of PTH levels in the vitamin D group. Thus, in general, we consider this RCT as suitable for evaluating vitamin D effects on BTMs.

## 5. Conclusions

In summary, we found no significant vitamin D effect on BTMs or BMD in this cohort. This finding confirms previous studies and suggests that vitamin D treatment has no clinically relevant short-term effect on bone turnover and BMD in these middle-aged healthy men with baseline 25(OH)D levels <75 nmol/L. Future RCTs should be performed in vitamin D deficient subjects. Further, future trials should consider obesity when selecting an optimal vitamin D dose, as vitamin D was effective in reducing PTH levels only in normal weight subjects.

## Figures and Tables

**Table 1:** Baseline characteristics of study participants.

	All Study Participants (*n* = 200)		Vitamin D (*n* = 100)		Placebo (*n* = 100)		
	Median	IQR	Median	IQR	Median	IQR	*p*-Value
Age (years)	45	31–54	40	30–53	47	32–55	0.230
BMI (kg/m^2^)	26.4	24.1–29.5	26.3	24.0–29.3	26.8	24.1–29.8	0.548
Total testosterone (nmol/L)	15.8	12.3–18.9	15.8	12.3–19.4	15.8	12.3–18.3	0.443
25-hydroxyvitamin D (nmol/L)	52.5	42.0–67.5	53	43–68	52	42–64	0.847
PTH (pg/mL)	45.8	35.8–55.6	46.6	36.2–57.0	43.4	35.8–54.3	0.317
Serum calcium (mmol/L)	2.38	2.33–2.43	2.37	2.33–2.43	2.39	2.34–2.43	0.313
Serum phosphate (mmol/L)	0.90	0.78–1.04	0.92	0.78–1.06	0.88	0.78–1.01	0.910
Urine calcium (mmol/L)	2.65	1.39–3.84	2.57	1.37–3.86	2.75	1.40–3.84	0.890
Osteocalcin (ng/mL)	21.1	17.0–27.1	20.9	16.7–25.5	21.5	17.6–27.8	0.449
CTX (ng/mL)	0.37	0.27–0.50	0.40	0.27–0.50	0.35	0.27–0.50	0.994
Total body BMD (g/cm^2^)	1.299	1.230–1.366	1.294	1.221–1.369	1.299	1.234–1.360	0.574
Spine BMD (g/cm^2^)	1.230	1.108–1.326	1.233	1.106–1.334	1.213	1.110–1.315	0.657
Femoral neck BMD (g/cm^2^)	1.023	0.941–1.125	1.027	0.932–1.143	1.022	0.956–1.120	0.346
Total hip BMD (g/cm^2^)	1.076	1.001–1.168	1.073	0.993–1.184	1.076	1.002–1.156	0.629
TBS	1.368	1.313–1.463	1.357	1.313–1.521	1.382	1.351–1.445	0.743
Season 1 (%)	34.0		32		36		0.386
Season 2 (%)	37.5		34		41		
Season 3 (%)	11.5		14		9		
Season 4 (%)	17.0		20		14		

Comparisons of baseline characteristics between men in the vitamin D and the placebo group were performed using student’s *t*-test and χ^2^-test. Season 1: January–March, season 2: April–June, season 3: July–September, season 4: October–December; IQR=interquartile range, PTH = parathyroid hormone, CTX = beta-crosslaps, BMD = bone mineral density, TBS = trabecular bone score.

**Table 2:** Parameters of mineral metabolism and outcome variables at baseline and final follow-up at study end (12 weeks) in study participants with available values at both study visits.

	Baseline Visit		Study End		Treatment Effect		
	Median	IQR	Median	IQR	Between Group Differences with 95% CI		*p*-Value
**Mineral metabolism**							
25-hydroxyvitamin D (nmol/L)							
Vitamin D (*n* = 96)	53	43–68	98	85–116	37.4	31.3 to 43.6	<0.001
Placebo (*n* = 96)	52	42–64	65	51–77			
PTH (pg/mL)							
Vitamin D (*n* = 94)	46.6	36.2–57.0	46.4	35.0–59.2	−4.8	−8.9 to −0.8	0.021
Placebo (*n* = 95)	43.4	35.8–54.3	49.3	38.7–61.6			
**Bone turnover markers**							
CTX (nmol/L)							
Vitamin D (*n* = 94)	0.40	0.27–0.50	0.36	0.23–0.48	−0.01	−0.04 to 0.03	0.792
Placebo (*n* = 96)	0.35	0.27–0.50	0.36	0.27–0.45			
Osteocalcin (ng/mL)							
Vitamin D (*n* = 94)	20.9	16.7–25.5	21.5	17.6–27.8	0.84	−0.76 to 2.44	0.300
Placebo (*n* = 96)	21.2	16.8–24.3	20.3	17.7–23.9			
**BMD**							
Total body BMD (g/cm^2^)							
Vitamin D (*n* = 94)	1.294	1.221–1.369	1.300	1.239–1.366	0.006	−0.006 to 0.020	0.293
Placebo (*n* = 92)	1.299	1.234–1.360	1.295	1.225–1.368			
Lumbar spine BMD (g/cm^2^)							
Vitamin D (*n* = 94)	1.233	1.106–1.334	1.227	1.100–1.345	0.002	−0.009 to 0.012	0.760
Placebo (*n* = 92)	1.213	1.110–1.315	1.219	1.099–1.326			
Femoral neck BMD (g/cm^2^)							
Vitamin D (*n* = 94)	1.027	0.932–1.143	1.037	0.928–1.141	0.002	−0.006 to 0.011	0.637
Placebo (*n* = 92)	1.022	0.956–1.120	1.012	0.943–1.113			
Total hip BMD (g/cm^2^)							
Vitamin D (*n* = 94)	1.073	0.993–1.184	1.075	0.984–1.177	−0.002	−0.009 to 0.006	0.662
Placebo (*n* = 92)	1.076	1.002–1.156	1.067	1.002–1.165			
TBS							
Vitamin D (*n* = 53)	1.357	1.313–1.521	1.375	1.273–1.484	0.037	−0.102 to 0.176	0.596
Placebo (*n* = 55)	1.382	1.351–1.445	1.378	1.319–1.418			

IQR = interquartile range, PTH = parathyroid hormone, CTX = beta-crosslaps, BMD = bone mineral density, TBS = trabecular bone score. Data are shown as medians and interquartile range. Treatment effects with 95% confidence interval and *p*-values were calculated by ANCOVA for group differences at follow-up with adjustment for baseline values.

**Table 3:** Parameters of mineral metabolism at baseline and final follow-up at study end (12 weeks) in various subgroup analyses (study participants with baseline 25(OH)D levels <40 nmol/L and with baseline 25(OH)D levels ≥40 nmol/L; study participants with a baseline body mass index (BMI) <25 kg/m^2^ and with a baseline BMI ≥25 kg/m^2^; study participants with baseline total testosterone levels <10.41 nmol/L and with a baseline total testosterone levels ≥10.41 nmol/L) with available values at both study visits.

	Baseline Visit		Study End		Treatment Effect		
	Median	IQR	Median	IQR	Between Group Differences with 95% CI		*p*-Value
**Men with baseline 25-hydroxyvitamin D levels <40 nmol/L**							
25-hydroxyvitamin D (nmol/L)							
Vitamin D (*n* = 19)	34	30–37	86	74–107	45.2	29.4 to 64.4	<0.001
Placebo (*n* = 16)	29	23–36	38	27–60			
Parathyroid hormone (pg/mL)							
Vitamin D (*n* = 19)	56.0	39.8–71.4	47.4	37.3–67.2	2.3	−7.3 to 12.0	0.625
Placebo (*n* = 16)	47.8	42.2–63.0	47.2	40.0–53.4			
**Men with baseline 25-hydroxyvitamin D levels ≥40 nmol/L**							
25-hydroxyvitamin D (nmol/L)							
Vitamin D (*n* = 77)	58	49–71	105	86–116	35.5	28.8 to 42.2	<0.001
Placebo (*n* = 80)	57	48–69	68	55–79			
Parathyroid hormone (pg/mL)							
Vitamin D (*n* = 75)	45.6	36.2–54.4	45.4	33.9–55.9	−6.4	−11.0 to −1.9	0.005
Placebo (*n* = 79)	42.8	35.8–52.4	49.6	38.5–62.2			
**Men with a baseline BMI <25 kg/m^2^**							
25-hydroxyvitamin D (nmol/L)							
Vitamin D (*n* = 30)	56	44–70	110	86–119	43.8	31.7 to 55.9	<0.001
Placebo (*n* = 32)	48	38–64	72	36–80			
Parathyroid hormone (pg/mL)							
Vitamin D (*n* = 30)	46.6	33.7–54.0	41.6	31.5–50.1	−9.1	−17.4 to −0.8	0.033
Placebo (*n* = 32)	40.2	30.6–48.8	46.0	35.1–64.8			
**Men with a baseline BMI ≥25 kg/m^2^**							
25-hydroxyvitamin D (nmol/L)							
Vitamin D (*n* = 66)	52	42–68	54	43–67	34.9	27.7 to 42.0	<0.001
Placebo (*n* = 64)	95	84–112	63	54–76			
Parathyroid hormone (pg/mL)							
Vitamin D (*n* = 64)	46.7	37.9–61.1	48.7	38.4–62.7	−3.0	−7.6 to 1.6	0.199
Placebo (*n* = 63)	45.0	37.2–56.7	51.8	40.9–61.0			
**Men with baseline total testosterone levels <10.41 nmol/L**							
25-hydroxyvitamin D (nmol/L)							
Vitamin D (*n* = 47)	56	44–72	89	83–110	32.3	23.3 to 41.3	<0.001
Placebo (*n* = 47)	53	42–63	62	52–76			
Parathyroid hormone (pg/mL)							
Vitamin D (*n* = 46)	45.4	35.2–58.0	48.6	37.3–60.2	−0.9	−5.7 to 4.0	0.727
Placebo (*n* = 47)	43.1	36.3–55.2	49.9	37.1–58.2			
**Men with baseline total testosterone levels ≥10.41 nmol/L**							
25-hydroxyvitamin D (nmol/L)							
Vitamin D (*n* = 49)	52	42–65	107	89–119	42.1	33.6 to 50.6	<0.001
Placebo (*n* = 49)	51	43.68	69	46–79			
Parathyroid hormone (pg/mL)							
Vitamin D (*n* = 48)	49.5	37.0–55.6	45.0	33.9–53.2	−9.8	−15.3 to −2.2	0.009
Placebo (*n* = 48)	44.5	35.8–50.2	48.6	39.9–65.4			

Data are shown as medians and interquartile range. Treatment effects with 95% confidence interval and *p*-values were calculated by ANCOVA for group differences at follow-up with adjustment for baseline values. IQR = interquartile range.

**Table 4:** Parameters of mineral metabolism and outcome variables at baseline and final follow-up at study end (12 weeks) in study participants with a BMI <26.4 kg/m^2^ and available values at both study visits.

	Baseline Visit		Study End		Treatment Effect		
	Median	IQR	Median	IQR	Between Group Differences with 95% CI		*p*-Value
**Mineral metabolism**							
25-hydroxyvitamin D (nmol/L)							
Vitamin D (*n* = 50)	57	44–71	108	88–122	45.2	35.3 to 55.7	<0.001
Placebo (*n* = 47)	52	41–66	71	41–77			
PTH (pg/mL)							
Vitamin D (*n* = 50)	46.2	35.0–55.8	44.0	32.2–51.8	−8.1	−14.7 to −1.5	0.017
Placebo (*n* = 47)	43.0	35.8–50.2	49.2	37.4–63.7			
**Bone turnover markers**							
CTX (nmol/L)							
Vitamin D (*n* = 50)	0.42	0.32–0.52	0.38	0.25–0.41	0.01	−0.05 to 0.06	0.774
Placebo (*n* = 47)	0.42	0.27–0.59	0.38	0.30–0.44			
Osteocalcin (ng/mL)							
Vitamin D (*n* = 50)	21.8	18.4–28.1	22.5	20.5–28.1	0.46	−2.47 to 3.39	0.758
Placebo (*n* = 47)	23.5	18.6–28.1	22.8	18.5–24.9			
**BMD**							
Total body BMD (g/cm^2^)							
Vitamin D (*n* = 50)	1.271	1.190–1.346	1.281	1.203–1.347	0.012	0.001 to 0.023	0.033
Placebo (*n* = 47)	1.276	1.200–1.341	1.270	1.203–1.349			
Lumbar spine BMD (g/cm^2^)							
Vitamin D (*n* = 50)	1.218	1.077–1.306	1.187	1.051–1.287	−0.004	−0.019 to 0.011	0.612
Placebo (*n* = 47)	1.209	1.098–1.286	1.197	1.092–1.294			
Femoral neck BMD (g/cm^2^)							
Vitamin D (*n* = 50)	1.023	0.918–1.131	1.027	0.910–1.127	−0.018	−0.065 to 0.028	0.438
Placebo (*n* = 47)	1.029	0.948–1.146	1.013	0.935–1.136			
Total hip BMD (g/cm^2^)							
Vitamin D (*n* = 50)	1.037	0.966–1.168	1.070	0.960–1.164	0.020	−0.089 to 0.129	0.716
Placebo (*n* = 47)	1.046	0.984–1.132	1.032	0.968–1.133			
TBS							
Vitamin D (*n* = 23)	1.357	1.313–1.521	1.375	1.273–1.484	−0.003	−0.191 to 0.185	0.947
Placebo (*n* = 22)	1.382	1.351–1.445	1.378	1.319–1.418			

IQR = interquartile range, PTH = parathyroid hormone, CTX = beta-crosslaps, BMD = bone mineral density, TBS = trabecular bone score. Data are shown as medians and interquartile range. Treatment effects with 95% confidence interval and p-values were calculated by ANCOVA for group differences at follow-up with adjustment for baseline values.

## Supplementary Materials

The following are available online at https://www.mdpi.com/2072-6643/11/4/731/s1, Figure S1: 25(OH)D levels at baseline and after 12 weeks in the placebo group. Paired student’s T-test was used for comparisons of 25(OH)D levels at baseline and follow-up.
