# Supplementary material for: Effects of Vitamin D Supplementation on Bone Turnover and Bone Mineral Density in Healthy Men: A Post-Hoc Analysis of a Randomized Controlled Trial
Source: Nutrients. 2019 Mar 29;11(4):731. doi: 10.3390/nu11040731 (PMC6521067; doi:10.3390/nu11040731)

Supplemental figure 1: 25(OH)D levels at baseline and after 12 weeks in the placebo group. Paired student's T-test was used for comparisons of 25(OH)D levels at baseline and follow-up

Figure 1a: All subjects (n=96), p<0.001

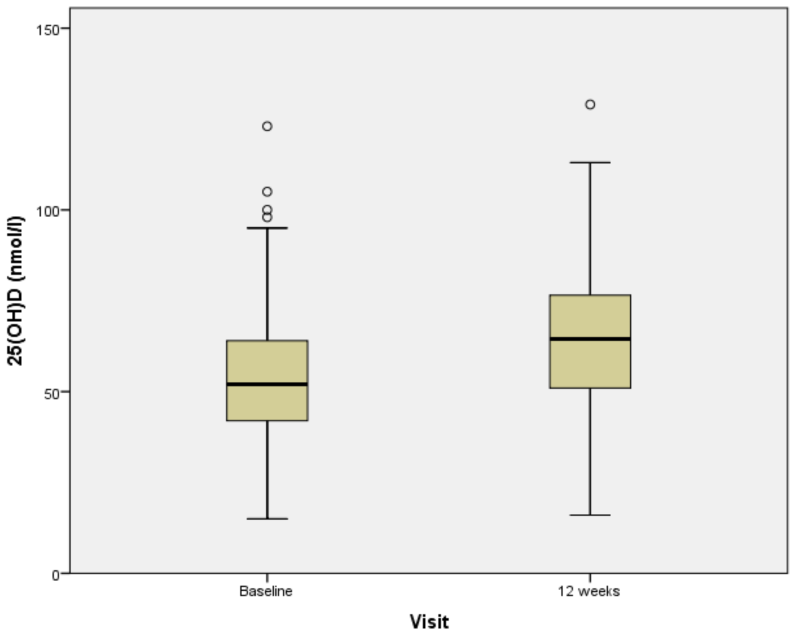

Figure 1b: Subjects with a baseline 25(OH)D level <40 nmol/l (n=16), p=0.051

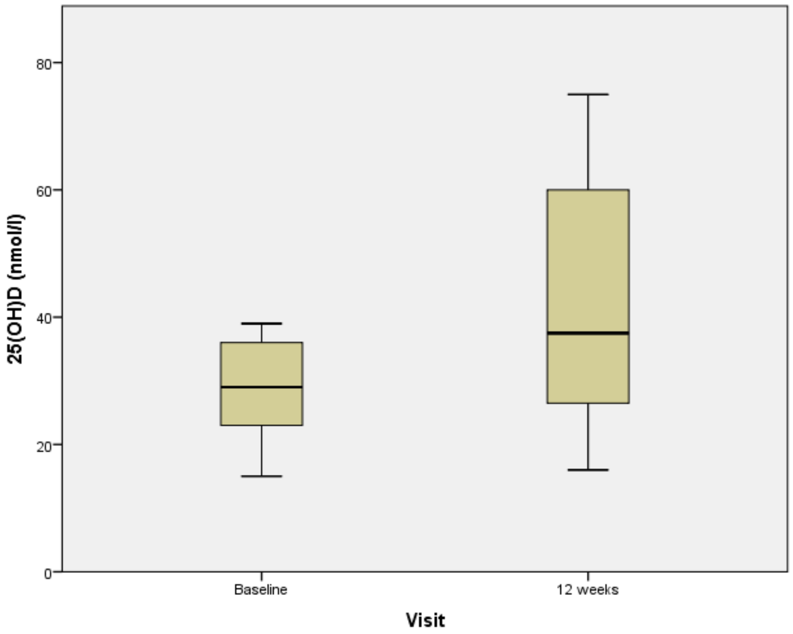

Figure 1c: Subjects with a baseline 25(OH)D level ≥40 nmol/l (n=80), $p<0.001$

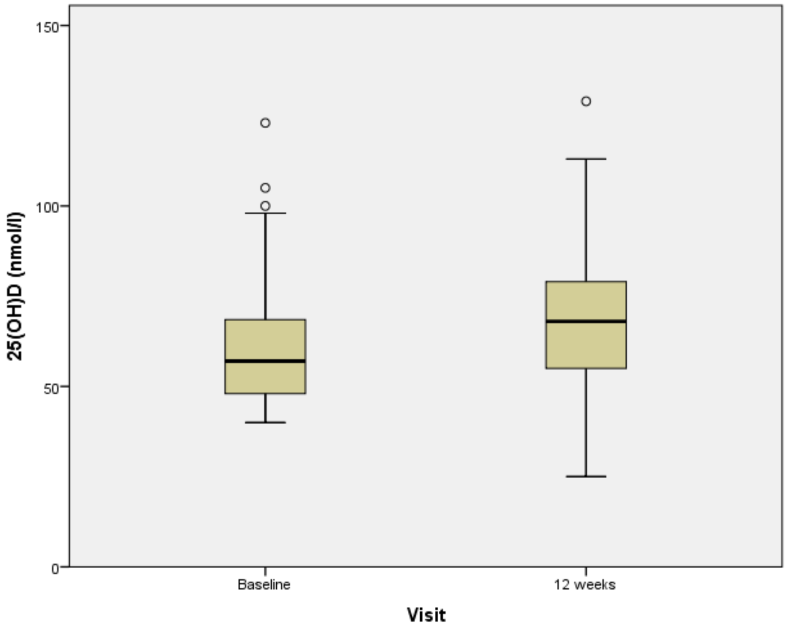

Figure 1d: Subjects with a BMI <25 kg/m² (n=32), $p=0.008$

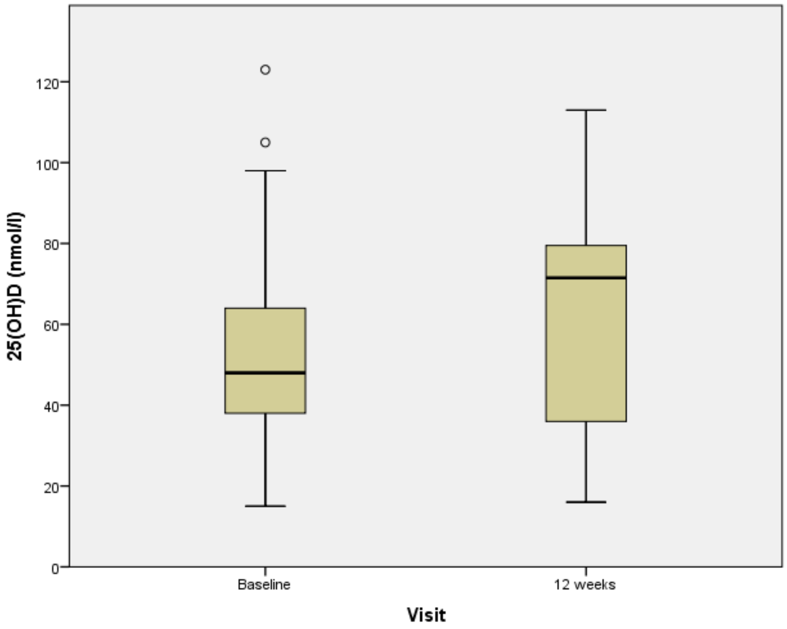

Figure 1e: Subjects with a BMI ≥25 kg/m² (n=64), p<0.001

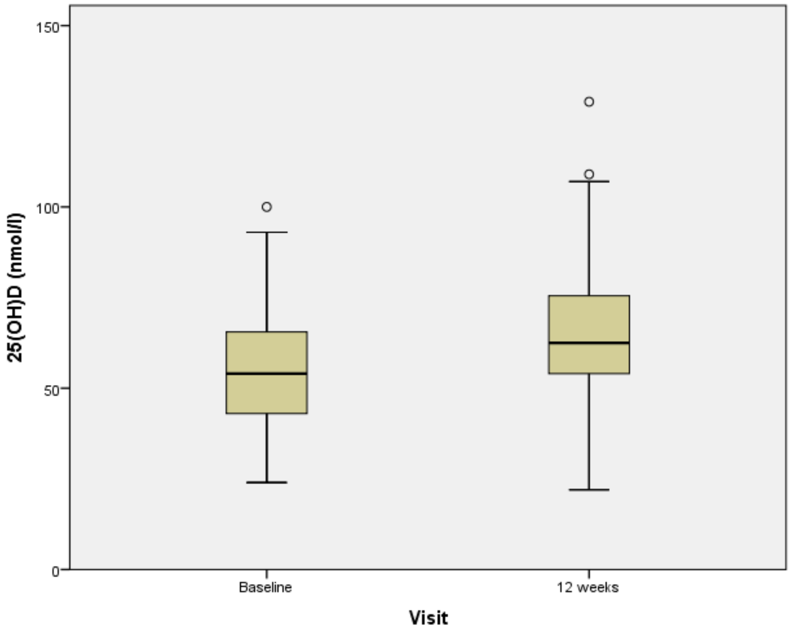

Figure 1f: Subjects with a baseline TT level <10.41 nmol/l (n=47), p<0.001

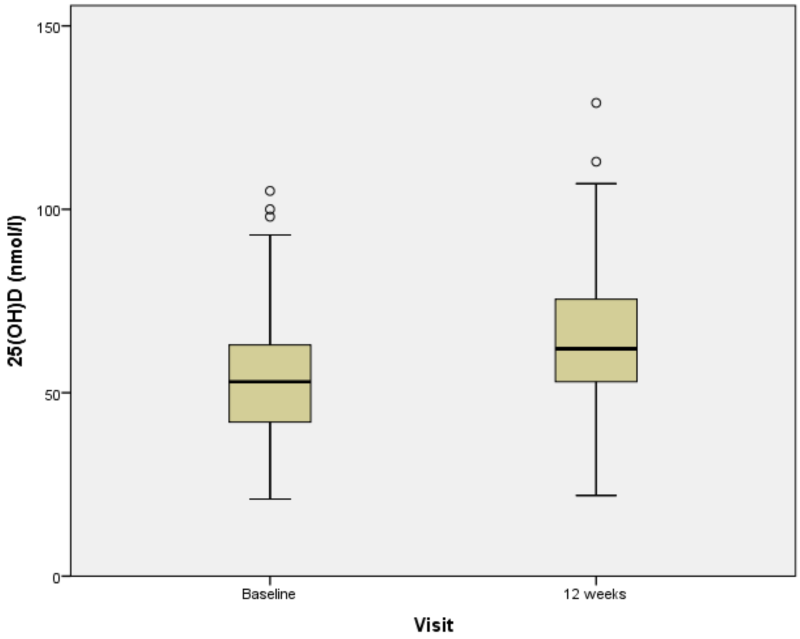

Figure 1g: Subjects with a baseline TT level ≥10.41 nmol/l (n=49), p=0.001

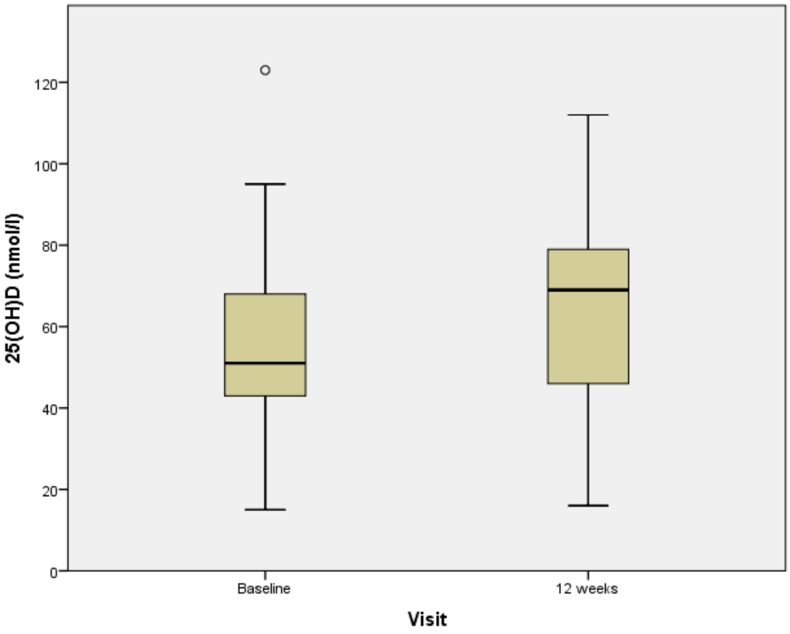

Supplement: Supplementary file 1 [file nutrients-11-00731-s001.pdf]
